# Rolling Contact Fatigue Performances of Carburized and High-C Nanostructured Bainitic Steels

**DOI:** 10.3390/ma9120960

**Published:** 2016-11-25

**Authors:** Yanhui Wang, Fucheng Zhang, Zhinan Yang, Bo Lv, Chunlei Zheng

**Affiliations:** 1 State Key Laboratory of Metastable Materials Science and Technology, Yanshan University, Qinhuangdao 066004, China; wyh@stumail.ysu.edu.cn (Y.W.); zhengclysu@ysu.edu.cn (C.Z.); 2 National Engineering Research Center for Equipment and Technology of Cold Strip Rolling, Yanshan University, Qinhuangdao 066004, China; zhinanyang@ysu.edu.cn; 3 College of Environmental and Chemical Engineering, Yanshan University, Qinhuangdao 066004, China; lvbo@ysu.edu.cn

**Keywords:** nanostructured bainite, carbide, retained austenite, residual stress, rolling contact fatigue

## Abstract

In the present work, the nanostructured bainitic microstructures were obtained at the surfaces of a carburized steel and a high-C steel. The rolling contact fatigue (RCF) performances of the two alloy steels with the same volume fraction of undissolved carbide were studied under lubrication. Results show that the RCF life of the carburized nanostructured bainitic steel is superior to that of the high-C nanostructured bainitic steel in spite of the chemical composition, phase constituent, plate thickness of bainitic ferrite, hardness, and residual compressive stress value of the contact surfaces of the two steels under roughly similar conditions. The excellent RCF performance of the carburized nanostructured bainitic steel is mainly attributed to the following reasons: finer carbide dispersion distribution in the top surface, the higher residual compressive stress values in the carburized layer, the deeper residual compressive stress layer, the higher work hardening ability, the larger amount of retained austenite transforming into martensite at the surface and the more stable untransformed retained austenite left in the top surface of the steel.

## 1. Introduction

The phase transformation theories, composition design, microstructure characteristics, and conventional mechanical properties of nanostructured bainitic steels have been widely studied by numerous material researchers in the past decade [1,2,3,4,5,6,7,8,9,10]. The extraordinarily fine bainitic ferrite plates are responsible for the excellent strength, and the carbon-enriched retained austenite films account for the high toughness of the nanostructured bainitic steels. Most of these nanostructured bainitic steels are high-carbon (C) and high-silicon (Si) steels [1,2,3,7,8,9]. The high content of Si, which is usually more than 1.4 wt.% and has very low solubility in cementite, can greatly suppress the precipitation of cementite during bainitic transformation and retard its growth from austenite. The carbon that is rejected from the bainitic ferrite enriches the retained austenite, thereby stabilizing it down to ambient conditions. The resulting microstructure consists of fine plates of bainitic ferrite separated by carbon-enriched regions of austenite. Kim et al. [11] reported that the Si-modified 100Cr6 bearing steel is 3–4 times superior in terms of rolling contact fatigue (RCF) characteristics compared with the conventional steel when the Si content is increased from 0.25 wt.% to 1.50 wt.%. The excellent RCF performance is attributed to the reduced maximum size of the non-metallic inclusions and the increased stabilization of retained austenite due to the fact that no carbide precipitation occurs during tempering when a higher amount of Si was added to the steel.

In recent years, the fatigue performances of nanostructured bainitic steels have attracted a large number of material researchers. In 2011, Peet et al. [12] first studied the axial fatigue behavior of nanostructured bainitic steel which was smelted in air. The fatigue limit of ~855 MPa for no failure in 10^7^ cycles estimated by extrapolating data of the maximum cycle number of 10^5^ is generally consistent with published work on iron alloys of similar hardness. Subsequently, a study by Yang et al. suggests that the high-C Si–Al-rich nanostructured bainitic steel has an outstanding high-cycle bending fatigue performance, whose fatigue limits for no failure in 10^7^ cycles are 1033–1156 MPa [13]. They presented that the plastic deformation and martensitic transformation of the retained austenite under stress or strain can blunt the crack by absorbing more energy than necessary for fatigue crack propagation. The nanostructured bainitic microstructure, retained austenite, and secondary cracks resulting from crack branching on ferrite/austenite interfaces, rather than just the hardness or strength, play an important role in improving the fatigue performance. Recently, an investigation on rotating bending fatigue behavior of nanostructured low-temperature bainitic steel was performed on a 0.76 wt.% C steel [14]. Fatigue strength has been shown to be increased by reducing the bainitic transformation temperature where fatigue limits were measured to be 820, 945 and 1005 MPa for the samples isothermally transformed at 300, 250 and 200 °C, respectively. They observed that shear strain and secondary crack initiation seem to occur inside the austenite phase for the samples transformed at 300 °C because of a lower C content and lower stability of the austenite in the center of this phase. In addition, the nanostructured bainitic microstructure, which was obtained in the surface layer of low-carbon steel based on carburization and subsequent low-temperature austempering, achieved a service life about twice that of the traditional martensite carburizing steel, exhibiting an excellent resistance to RCF [15]. Studies have shown that traditional 1C–1.5Cr bearing steel with the lower bainite microstructure exhibits an enhanced RCF performance in a contaminated lubricating environment [16,17]. Solano-Alvarez et al. [18] have studied the RCF phenomena and proposed the damage mechanism of a nanostructured bainitic steel without undissolved carbides. They pointed out that the degradation mechanism is ductile void formation at the interfaces, followed by growth and coalescence into larger voids that cause fracture along the direction of the softer phase. This mechanism is similar to the crack formation mechanism during RCF in Hadfield steel crossing [19] but different from the conventional damage mechanism, which involves crack initiation at inclusions and propagation in typical bearing steels. However, the damage mechanism of the nanostructured bainitic steel with undissolved carbides is still an interesting topic and needs to be further studied. Liu et al. [20] have recently reported that the RCF life of an ultrahigh carbon steel with a mixed microstructure composed of nanobainite, martensite, retained austenite, and undissolved spherical carbides is approximately 3.3 times longer than that of the steel with tempered martensite. The improvement in the RCF life of the steel is attributed to nanobainite and stable film-like retained austenite. The nanobainite can delay crack initiation by alleviating the stress concentration at the hard phases (such as non-metal inclusions or carbide particles), and the stable film-like retained austenite can retard crack propagation. These research findings provide a theoretical basis and technical support for the eventual usage of nanostructured bainitic steels in the rolling bearing field.

RCF is a very complicated failure process, and the RCF life of steels is the result of the synergistic effect of many factors [17]. In recent years, with the development of steelmaking technology, the size and amount of inclusions are greatly reduced by the successful development of high-purity steel with low oxygen content. The number of inclusions with the diameter bigger than 10 µm in 1 cm^3^ was reduced from 6 × 10^5^ to 6 × 10^4^ when the oxygen content was reduced from 50 ppm to 5 ppm in the steel [21]. With this background, studying the other influencing factors on RCF life from the point of view of the material itself is significant. In the present study, the RCF performances of carburized and high-C nanostructured bainitic steels were comparatively studied from various aspects, such as residual stress distribution, undissolved carbide morphology, hardness value, and retained austenite content of the two steels.

## 2. Experimental Procedure

**Figure 1:**
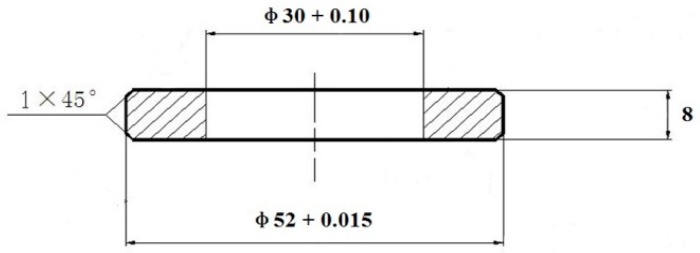
Sketch of RCF test specimen, (mm).

The carburized steel and high-C steel, which were designated as CC steel and HC steel in this paper, respectively, were smelted with an arc-furnace followed by electroslag remelting. Their chemical compositions are listed in Table 1. First, the CC steel was machined into the specimens for RCF tests according to Figure 1. Then, the specimens were carburized. The carburizing process and the carbon content distribution from the top surface to the center are the same as those in [22]. The carbon content at the top surface was determined to be ~0.88 wt.% using a spectrometer model PAD-5500 II. Based on the following equation [23], the martensite-start (*M*_s_) temperatures of the core and the surface of the CC steel were calculated to be ~371 and ~150 °C, respectively. The *M*_s_ temperature of the HC steel was calculated to be ~168 °C.

*M_s_* = 512 − {453 × (%C)} − {16.9 × (%Ni)} + {15 × (%Cr)} − {9.5 × (%Mo)} + {217 × (%C)^2^} − {71.5 × (%C) × (%Mn)} − {67.6 × (%C) × (%Cr)} (1)

After carburizing, the specimens of the CC steel were tempered at 650 °C for 3 h, which is in fact a spheroidizing annealing process and the carbides would be precipitated in the microstructure of the surface during the tempering process. Then, the specimens were austenitized at 860 °C for 1 h and austempered at 200 °C for 8 h in molten salt composed of sodium nitrite and potassium nitrate (1:1 in weight).

For the HC steel, the specimens for the RCF tests were machined from the spheroidized annealed steel bars. The spheroidizing annealing process is the same as that used in [24]. Then, the specimens were austenitized at 860 °C for 1 h and austempered at 210 °C for 4 h in the molten salt. Finally, all specimens of the two steels were tempered at 200 °C for 1 h to relieve the quenching stress and improve the toughness of the microstructure.

The CC steel after carburizing and all the HC steel specimens were sealed with an anti-oxidation and anti-decarburization protective coating at high temperature (500 °C–1200 °C) model KOC-15 to prevent decarburization during heat treatment. After the above heat treatments, almost the same hardness values of 730 ± 10 HV0.5 and 727 ± 8 HV0.5 were obtained at the surfaces of the CC steel and the HC steel, respectively.

**Figure 2:**
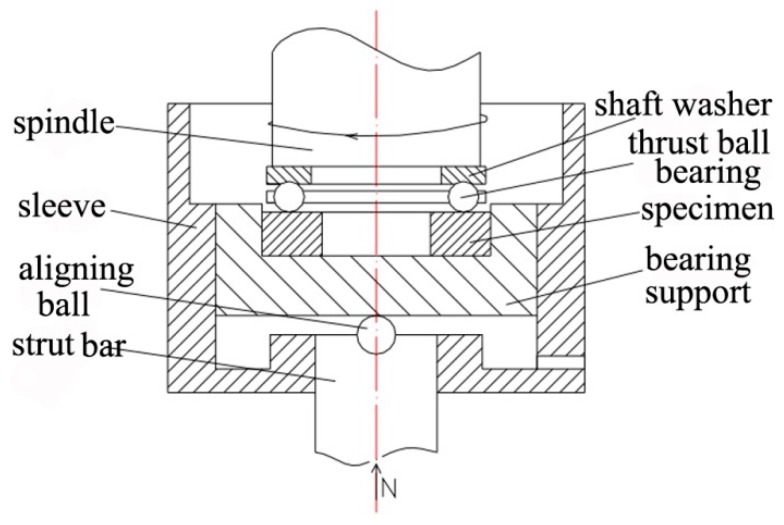
Sketch of RCF testing principle.

Before the RCF test, the surfaces of all specimens were ground with SiC abrasive paper and polished with a diamond paste model W 0.5 µm. The RCF test was carried out with a point contact RCF testing machine model TLP-1. All tests were performed at a rotational speed of 2040 rpm and a Hertzian pressure of 4500 MPa. The N32 mechanical oil was used as lubricant at room temperature. The oil was filtered by a filter model QU-A10 × 10 with the filter element model 7ZX2-10 × 10Q. The oil was individually recirculated for each specimen. The sketch of the RCF testing principle in this study is shown in Figure 2. Vibration levels were monitored through an accelerometer, which automatically stopped tests if the thresholds were surpassed, caused normally only by flaking or spalling. Data of the RCF lives of 12 specimens for each steel were acquired and used in a Weibull distribution.

The microstructure observations were carried out using scanning electron microscopy (SEM, Hitachi-4800) and transmission electron microscopy (TEM, JEM-2010). The foils of CC steel for TEM observation were ground to ~30 µm thickness and acquired only from the backside of the carburization surface using different SiC abrasive papers to ensure that the top surface of the carburization layer was investigated. The foils of HC steel for TEM observation were also ground to ~30 µm. Then, all the foils were thinned to perforation on a TenuPol-5 twin-jet unit with an electrolyte consisting of 7 vol.% perchloric acid and 93 vol.% glacial acetic acid. The volume fraction of retained austenite of the surface after different RCF testing cycles was analyzed on a Brucker-D8 Advance™ micro-region X-ray diffractometer (XRD) system with Cu *K*_α_ radiation at 40 kV and 40 mA. The angular resolution of the D8 Advance XRD is less than 0.037°. The diffraction profiles were obtained by varying 2*θ* from 35° to 105° with a step size of 0.02°. The time spent collecting the data per step was 2.4 s. The specimens for XRD analysis were carefully prepared and the surfaces were carefully protected. The surfaces of the specimens were ultrasonically cleaned with ethanol after the RCF test, and dried by a blower. Then, the smoother area within the contact track was chosen for the XRD test. The residual stress distribution with depth from the surface of the specimen before RCF testing was measured by an X-350A X-ray stress meter using Cr *K*_α_ radiation at 28 kV and 8 mA. Scans were performed from 146° to 168°, with a step size of 0.05° and a dwell time of 0.5 s. The classical sin^2^*ψ* method was applied to evaluate the stress by using *ψ* = 0° and 45° for the 211 reflection, which corresponds to the ferrite phase. The microhardness of the surface and microhardness distribution just below the rolling contact surface of the specimen after different RCF testing cycles were measured using the Vickers tester model HVS-1000 under an applied load of 500 g and a dwelling time of 10 s. The specimen for microhardness distribution testing was cut out along the axial direction, which is perpendicular to the plane that the rolling contact surface locates. Then, the cross-section was ground with SiC abrasive paper and polished with diamond paste. The hardness values at different depths were obtained just below the rolling contact surface on the cross-section.

## 3. Results and Discussion

**Figure 3:**
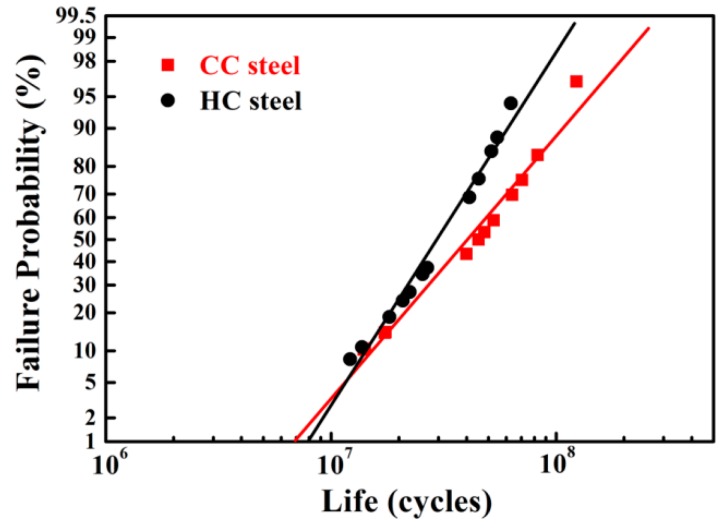
Weibull plots of RCF lives of CC and HC steels.

Figure 3 shows the Weibull plots of RCF lives of CC and HC steels, and Table 2 summarizes the Weibull statistic results, such as the Weibull slope, *L*_10_ life, *L*_50_ life, and characteristic life. It can be clearly seen that the *L*_10_ life of the CC steel is approximately the same with that of the HC steel, but the *L*_50_ life and characteristic life of the CC steel are 1.4 times and 1.5 times longer than that of the HC steel, respectively.

**Figure 4:**
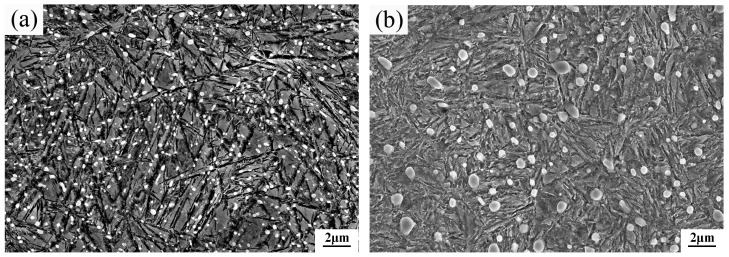
SEM images of the surface of the specimen after heat treatment: (**a**) CC steel and (**b**) HC steel.

Figure 4 shows the typical SEM images of the top surface of the two steels. Fine undissolved carbide particles were dispersed in the matrix of the two steels, which can improve the wear resistance of the steel [5]. Most of the carbides have a spherical shape, and only a few are irregularly shaped. The average volume percentages of the undissolved carbides in the surfaces of CC steel and HC steel were estimated to be 6.5% ± 0.4% and 6.8% ± 0.2%, respectively, through extensive SEM observations and measurements by Image-Pro-Plus software. In addition, all the carbides were assumed to be spherical to assess the average equivalent diameters. Then, the average equivalent diameters of the carbides of CC steel and HC steel were estimated to be 0.25 ± 0.02 and 0.48 ± 0.03 µm, respectively. The volume percentages of the undissolved carbides in the surfaces of the two steels are almost the same, yet a significant difference between the sizes of the carbides was observed.

The undissolved carbide is a hard and brittle phase in steel. Under the action of alternating stress, stress concentration easily occurs at the sites where the undissolved carbides are located. Thus, the undissolved carbides would become the source of fatigue crack, and the fatigue life is reduced. The undissolved carbides in bearing steels must be fine, spherical, and uniform in distribution in the matrix to prevent reduction of the RCF life [17,25,26]. In fact, the carbon concentration in the location near the undissolved carbide is different from that in the location remote from the undissolved carbide in the steel [27]. The carbon concentration difference is great when the carbide particles are large, and the carbon concentration difference is small when the carbide particles are small. The large carbon concentration difference in the matrix reduces the average RCF life of the steel [27]. Also, the outline of the smaller carbide is rounder, and the roundness of the larger carbide is poor [27]. A large number of carbides were employed to measure the ratio of the long axis to the short axis. The smaller the ratio, the rounder the carbide. The results showed that the ratios were 1.34 ± 0.12 for the carbides in the surface of CC steel and 1.63 ± 0.13 for the carbides in the surface of HC steel, which is consistent with [27]. Generally, stress concentration easily occurs at the sharp edges or uneven edges of the larger carbide, whose roundness is poor. As a result, micro-crack initiation occurs, and the toughness and RCF life of the steel is reduced. Furthermore, the bonding interfaces between the finer carbides and the matrix are less than those between the larger carbides and the matrix. Thus, the matching degree of the finer carbide with the matrix is higher than that of the larger carbide with the matrix. Therefore, stress concentration is harder to induce when the finer carbides are dispersed in the matrix, and it is more difficult for the finer carbides to become the source of fatigue crack, resulting in spalling. To sum up, the much finer undissolved carbides’ dispersion distribution in the top surface of the CC steel is very beneficial in improving the RCF life.

**Figure 5:**
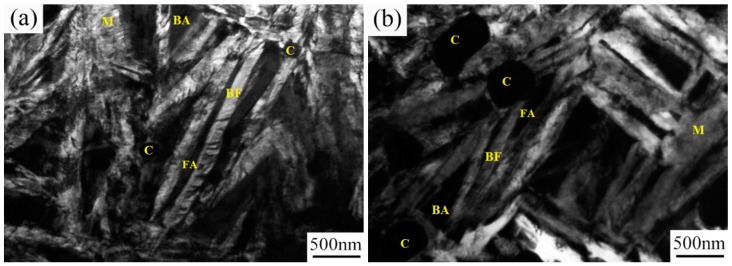
TEM micrographs of the surface of the specimen after heat treatment: (**a**) CC steel and (**b**) HC steel. Notes: BF—bainitic ferrite, FA—film-like austenite, BA—blocky austenite, M—martensite, and C—carbides.

The TEM micrographs of the surfaces of the two steels after the heat treatments are presented in Figure 5. The figure shows that a multiphase microstructure composed of nanostructured bainite, martensite, undissolved spherical carbides, and retained austenite were obtained at the top surface of the two steels after austempering. Carbide precipitation did not occur within the bainitic ferrite plate of the two steels as indicated by a large number of TEM observations. This is mainly associated with high Si content of the steels, which prevents cementite precipitation during bainitic transformation. A report has shown that adding 1.2–1.5 wt.% Si in bearing steel can inhibit carbide precipitation during low-temperature austempering [24]. From the TEM micrographs, the actual plate thickness of the bainitic ferrite (*t_BF_*) can be determined by measuring the mean lineal intercept ${\bar{L}}_{T}$ in a direction normal to the plate length and stereologically correcting the value in terms of ${\bar{L}}_{T}$ = *πt*/2 [28]. The measured mean thicknesses (*t_BF_*) of plates in the CC steel and the HC steel were about 68 ± 11 and 80 ± 14 nm, respectively. The formation of nanostructured bainite in the surface layers of the two steels is due to the high C and Si content, together with the very low phase transformation temperature [2,3,7,9].

As compared with martensite and carbide, the nanostructured bainite is a ductile phase in the steel, which is beneficial in improving the RCF performance, because it can alleviate stress concentration at the hard phases and relax the stress located at the crack tip [20]. Thus, the initiation and propagation of fatigue cracks would be hindered. The TEM photographs of the two steels showed that there were both blocky retained austenite and film-like retained austenite between the bainitic ferrite plates existing in the microstructure. The blocky retained austenite is metastable and transforms into martensite under stress, whereas the film-like retained austenite is relatively stable [29,30].

**Figure 6:**
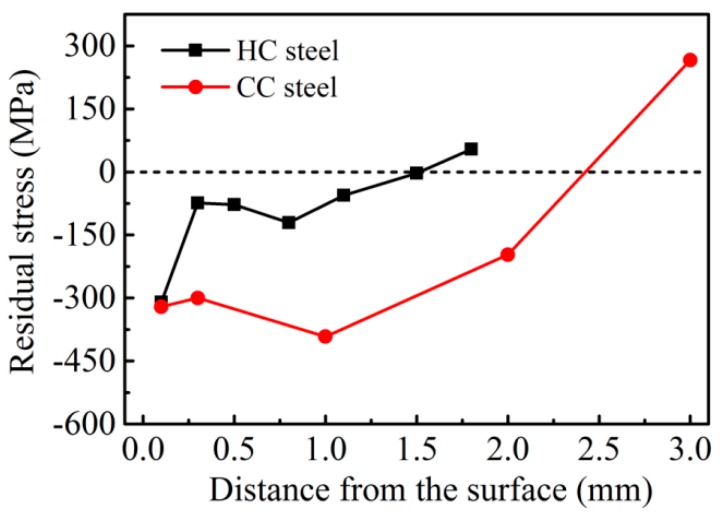
Residual stress distributions of the two steels after heat treatment. Notes: Residual stress at various depths was measured by an X-350A X-ray stress meter by means of the electrochemical corrosion layer stripping and electropolishing method.

Figure 6 shows the residual stress distributions of the two steels before the RCF test. The residual stresses in the surfaces of the two steels are both compressive stress, and the stress values are almost similar. Except for the top surface layer, the stress values in the compressive stress layer of the CC steel are much higher than that of the HC steel. During the RCF testing, the maximum shear stress was located below the surface. The depth of the maximum shear stress was calculated to be 0.263 mm below the contact surface based on the following equation [31]:

Z = 0.786b(2)

where b is the length of the short half axis of the contact ellipse. From Figure 6, we can work out that the residual stresses at the depth of 0.263 mm are −300 MPa and −74 MPa for CC steel and HC steel, respectively. The much higher residual compressive stress at the depth of 0.263 mm below the contact surface of the CC steel can alleviate the maximum shear stress more effectively. Thus, the RCF life of the steel is improved. The depths of the residual compressive stress layers of the CC steel and the HC steel are about ~2.5 and ~1.5 mm, respectively. The deeper residual compressive stress layer of the CC steel is caused by two important factors. One is the chemical composition difference between the carburizing layer and the core of the steel after carburizing. The other reason is that the phase transformation of the carburizing layer lags behind the martensitic transformation of the core when the CC steel is austempered at 200 °C [22]. The austempering process at 200 °C, which is much lower than the *M*_s_ temperature of the core and higher than the *M*_s_ temperature of the surface, ensures that the martensite transformation first occurs in the core. Because of the almost same residual compressive stress in the surfaces of the two steels, the differences in RCF life are mainly due to the depth of the residual compressive stress layer and the stress value level in the compressive stress layer.

It is well known that the tensile stress can help the crack open, and the compressive stress is helpful to the crack closure. Sadeghi [32] proposed that the cumulative fatigue damage rate is inversely proportional to the residual stress in the RCF test. Because the stress state of the surfaces of the two steels are both compressive stress (*σ_m_* < 0), the cumulative fatigue damage rate is decreased according to Sadeghi and the RCF life is prolonged. A report has shown [33] that the formation of the residual compressive stress layer in the steel can enhance the fatigue life by increasing the fatigue crack propagation threshold ∆*K*_th_ and reducing the fatigue crack growth rate *da*/*dN*. On one hand, the higher residual compressive stress value in the carburizing layer of CC steel causes the absorption of more energy for crack formation, thereby increasing the difficulty of crack initiation. On the other hand, the deeper residual compressive stress layer of CC steel greatly reduces the fatigue crack propagation rate in the carburizing layer, further improving the fatigue life.

**Figure 7:**
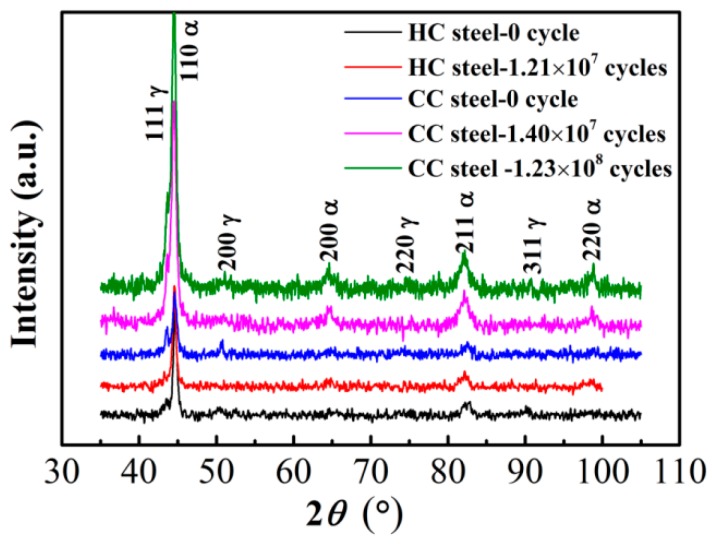
XRD patterns of contact surface of the two steels after RCF testing.

XRD patterns of the contact surface of the two steels after RCF testing are shown in Figure 7. In the patterns observed from the original surfaces (0 cycle) of the two steels, the peaks of ferrite (α) and austenite (γ) are visible. After different RCF testing cycles, the peaks of austenite of the two steels are weakened. The γ peaks are still visible in the CC steel. However, they nearly disappeared in the HC steel, thereby indicating that the volume fraction of the retained austenite under these conditions cannot be quantitatively calculated. The volume fraction of the retained austenite (*V*_γ_, vol.%) was determined according to Equation (3) in [34] using the 200, 211, and 220 reflections for the ferrite phase and the 200, 220 and 311 reflections for the austenite phase. The calculated results are shown in Table 3. Furthermore, the *V*_γ_ in the original surface of the HC steel is calculated to be 8.9 vol.%. These results indicate that there was retained austenite transforming into martensite on the surface of the two steels during the RCF testing. The volume fraction of retained austenite in the surface of the CC steel remained almost unchanged after 1.40 × 10^7^ cycles. The retained austenite on the surface of HC steel transforms almost completely after 1.21 × 10^7^ cycles. That is to say that the amount of retained austenite in the CC steel decreased by almost ~20% after the RCF testing whereas the HC steel lost almost all of the retained austenite. Therefore, we can infer that the untransformed retained austenite left in the surface of the CC steel after the RCF testing is very stable and its amount is about 12.4% ± 2.2%, which is much more than that of the HC steel.

The amount of the retained austenite transforming into martensite at the surface of the CC steel is much larger than that of the HC steel. On one hand, a compressive stress is produced when the retained austenite in the surface transforms into martensite because of volume expansion [17]. A greater amount of retained austenite transformed into martensite produces a greater compressive stress value. The greater residual compressive stress in the contact surface plays an important role in improving the RCF life. On the other hand, a higher amount of retained austenite that is transformed into martensite leads to absorption of more strain energy, which enhances the RCF life by delaying the crack initiation and propagation. However, the volume expansion caused by the retained austenite transforming into martensite significantly affects the dimensional stability of the bearing, especially for the precision bearing. The observed strain is about 10^−^^3^ per percent of retained austenite that decomposes in a 52,100 type steel [17].

Furthermore, more stable untransformed retained austenite left in the contact surface of the CC steel, which does not transform into martensite during the RCF testing, is very beneficial in improving the RCF life of the steel. First, the stable retained austenite in the contact surface can delay crack initiation via absorption of the plastic deformation energy generated by the contact stress. Second, the retained austenite, as a soft phase in the steel, increases the difficulty of crack initiation by alleviating the stress concentration at the hard phases (such as non-metal inclusions or carbide particles). Finally, the stress in the crack tip is relaxed when the tip of the fatigue crack encounters the soft phase of the retained austenite, thus more energy is needed to allow the crack to pass through the retained austenite. Therefore, the more stable untransformed retained austenite left in the surface of the CC steel can effectively prevent the crack initiation and propagation, which is different from the damage mechanism of the nanostructured bainitic steel without undissolved carbides in [18]. Thus, the RCF performance can be further improved.

**Figure 8:**
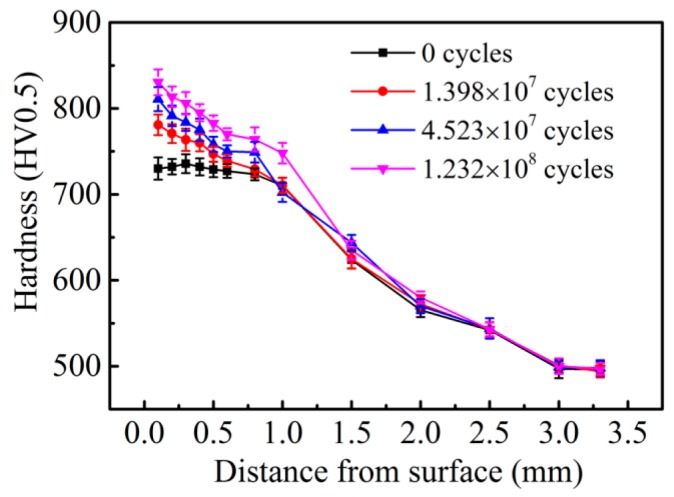
Hardness distributions of the CC steel after different RCF testing cycles.

**Figure 9:**
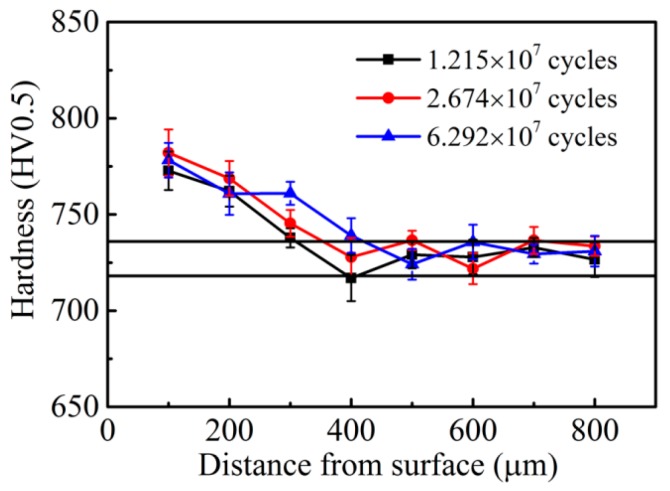
Hardness distributions of the HC steel after different RCF testing cycles (the two horizontal lines show the hardness ranges of the initial microstructure).

The hardness distributions of the CC steel and the HC steel after different RCF testing cycles are given in Figure 8 and Figure 9, respectively. The initial hardness values of the surfaces of the CC steel and the HC steel are 730 ± 10 HV0.5 and 727 ± 8 HV0.5, respectively. The hardness value of the contact surface of the CC steel increased significantly with increasing rolling cycles. The surface hardness value of the CC steel reached 840 ± 15 HV0.5 after rolling for 1.23 × 10^8^ cycles. However, the extent of the increase in surface hardness of the HC steel was significantly less than that of the CC steel after different rolling cycles. Moreover, the hardness values of the contact surface of the HC steel after different rolling cycles were nearly the same, all of which were within 780 ± 10 HV0.5. The depths of the hardened layer are about 1.0 mm for the CC steel and 0.4 mm for the HC steel, as shown in Figure 8 and Figure 9. The harder matrix of the HC steel prevents the continuous deformation of the subsurface layer during the RCF test, whereas the plastic deformation zone in the subsurface layer of the CC steel continuously enlarges because of the softer subsurface layer. The depth of the hardened layer is determined by the range of the plastic deformation zone during the RCF test. The increase in the contact surface hardness is mainly caused by the transformation of the retained austenite to martensite under the stress-strain effect during the RCF test. Bhadeshia [7] reported that the work-hardening capacity of the steel can be enhanced when the retained austenite undergoes stress- or strain-induced martensitic transformation. The work-hardening capacity of the CC steel is better than that of the HC steel because of the higher amount of retained austenite transforming into martensite in the surface and the better plasticity of the subsurface of the CC steel.

Surface hardness is often regarded as one of the criteria for evaluating the RCF life of steel. Zaretsky [35] proposed the empirical equation for the hardness value and the RCF life within a certain range of hardness value. The equation is expressed as follows:

(3) $$L_{10}^{2}/L_{10}^{1}=\mathrm{Exp}[m(R_{C2}-R_{C1})]$$

where $L_{10}^{2}$ and $L_{10}^{1}$ are the corresponding rated lives when the surface hardness values of the steels are *R*_C2_ and *R*_C1_, respectively, and *m* is a material constant with a value of 0.1. Because of the almost same surface hardness of the two steels, the value of $L_{10}^{\mathrm{CC}}$/$L_{10}^{\mathrm{HC}}$ is approximately 1 according to this empirical equation. However, the value of $L_{10}^{\mathrm{CC}}$/$L_{10}^{\mathrm{HC}}$ is approximately 1.03 according to the experimental results in Table 2. Therefore, we can conclude that the rated lives of the two steels are approximately the same based on the almost similar initial surface hardness, which is consistent with the experimental results in Table 2. Reports have shown [11,36] that the higher hardness of high-C–Cr bearing steel prolongs the RCF life. Thus, the RCF life of the specimen, which does not fail after 1.38 × 10^7^ cycles, is further increased by the hardness increasing. The work-hardening capacity directly depends on the degree of the hardness increase. The work-hardening capacity of the CC steel is significantly greater than that of the HC steel, which is one of the reasons for the longer median life and characteristic life of the CC steel than that of the HC steel. According to the above analysis, the RCF rating lives of the two steels mainly depend on the initial surface hardness values of the steels, whereas the median life and characteristic life are closely related to the work-hardening capacity of the steel during the RCF test.

## 4. Conclusions

The following conclusions can be drawn from the present work:

(1) The *L*_10_ life of the carburized nanostructured bainitic steel is approximately the same as that of the high-C nanostructured bainitic steel, but the *L*_50_ life and characteristic life of the former are 1.4 times and 1.5 times longer than those of the latter, respectively.
(2) The finer carbides’ dispersion distribution in the top surface, the higher residual compressive stress values in the carburized layer, the deeper residual compressive stress layer, the higher work-hardening capacity, the larger amount of retained austenite transforming into martensite at the surface and the more stable untransformed retained austenite left in the top surface play important roles in improving the RCF performance of carburized nanostructured bainitic steel.
(3) The RCF *L*_10_ lives of the carburized and high-C nanostructured bainitic steel mainly depend on the initial surface hardness values of the steels, whereas the *L*_50_ life and characteristic life are closely related to the work-hardening capacity of the steel during the RCF test.

## Figures and Tables

**Table 1:** Chemical compositions of the experimental steels (wt.%).

Steel	C	Si	Cr	Mo	Mn	Ni	P	S	O	Fe
CC	0.23	1.43	1.55	0.30	0.34	2.30	0.01	5 × 10^−3^	6 × 10^–4^	balance
HC	0.99	1.29	1.56	0.33	0.34	-	0.01	5 × 10^−3^	6 × 10^−4^	balance

**Table 2:** RCF lives of CC and HC steels.

Steel	*β*	*L*_10_ × 10^7^	*L*_50_ × 10^7^	*V*_s_ × 10^7^
CC	1.58	1.3751	4.5339	5.7143
HC	2.12	1.3342	3.2269	3.8354

Note: *L*_10_—rated life, *L*_50_—median life, *V*_s_—characteristic life, *β—*Weibull slope.

**Table 3:** The *V*_γ_ in the contact surface of the two steels after different RCF testing cycles.

Cycle (N)		0	1.21 × 10^7^	1.40 × 10^7^	1.23 × 10^8^
*V*_γ_ (vol.%)	CC steel	33.2 ± 1.5	-	13.0 ± 1.9	12.4 ± 2.2
	HC steel	8.9 ± 2.6	<2.0	-	-
